# Redefining Fitness in Chronic Lymphocytic Leukaemia: A Treatment-Specific Framework for the Targeted Therapy Era

**DOI:** 10.1159/ocl/aebag001

**Published:** 2026-08-27

**Authors:** Yousef Al-Asa’d, Salem H. Alshemmari, Nabeel Mohammad Qasem, Afaf Al Battah, Mohamed A. Yassin

**Affiliations:** a Department of Hematology, NCCCR, Hamad Medical Corporation, Doha, Qatar; b Department of Medicine, Department of Hematology, Kuwait Cancer Center, Faculty of Medicine, Kuwait University, Shuwaikh, Kuwait; c College of Medicine, Qatar University, Doha, Qatar

**Keywords:** Chronic lymphocytic leukaemia, Fitness, Frailty, Geriatric assessment, Bruton’s tyrosine kinase inhibitors, Venetoclax, Cardiovascular risk, Renal impairment, Fixed-duration therapy, Targeted therapy

## Abstract

**Background:**

Therapeutic advances in chronic lymphocytic leukaemia (CLL) have made treatment selection far more nuanced than in the chemoimmunotherapy era. Patients now have access to two principal therapeutic strategies: continuous therapy with Bruton’s tyrosine kinase inhibitors (BTKis) or fixed-duration regimens combining venetoclax with CD20 monoclonal antibodies and/or BTKis. These strategies carry fundamentally different safety profiles, monitoring requirements, and logistical demands, necessitating a treatment-specific approach to fitness assessment.

**Summary:**

The objective of this article was to synthesise current evidence and propose a practical, treatment-specific fitness assessment framework for patients with CLL, enabling physicians to match patient characteristics to the most appropriate targeted therapy. This was a narrative review of the literature from PubMed, Scopus, and Embase. Landmark randomised controlled trials, contemporary expert opinion papers, and current guidelines from ESMO, ASCO, and ESC were reviewed. The two therapeutic paradigms impose distinct fitness demands. For continuous BTKi therapy, cardiovascular comorbidity is the dominant determinant, given the class-effect risk of atrial fibrillation and hypertension. For fixed-duration venetoclax-based regimens, the key determinants instead shift to renal function and tumour burden, which govern tumour lysis syndrome risk, and to the social support and hospital access required for intensive ramp-up monitoring. Building on this distinction, the landmark phase 3 CLL17 trial (N Engl J Med, 2026) demonstrated that fixed-duration venetoclax-obinutuzumab and venetoclax-ibrutinib are non-inferior to continuous ibrutinib, with superior rates of undetectable minimal residual disease and lower treatment discontinuation, particularly in older patients. Notably, frailty – better conceptualised as lower resilience – is dynamic rather than fixed and may improve during effective targeted therapy. Structured pre-treatment screening can be performed with validated instruments such as the G8, VES-13, GAH, and the Canadian Study of Health and Aging Clinical Frailty Scale (CSHA-CFS); however, no single tool has yet been prospectively validated for outcome prediction specifically with BTKi or BCL-2 inhibitor therapy, and addressing this evidence gap is a priority for future research.

**Key Messages:**

A treatment-specific, individualised fitness assessment – rather than an age-based or aggregate comorbidity score – is the appropriate framework for CLL in the targeted therapy era. Most patients, including those with lower resilience, are candidates for targeted therapy; the clinical imperative is to match each patient to the treatment best suited to their specific fitness profile.

## Introduction

Chronic lymphocytic leukaemia (CLL) is the most prevalent haematological malignancy in Western countries, with a median age at diagnosis of approximately 72 years and an estimated 5-year relative survival now approaching 88.5%, a dramatic improvement from 65.1% in 1975 [1, 2]. This improvement is almost entirely attributable to the introduction of targeted agents, which have supplanted chemoimmunotherapy (CIT) as the standard of care across all patient groups [3].

Before fitness is considered, a diagnostic and baseline evaluation should be completed, as its results directly shape the treatment options that fitness assessment must then weigh. This baseline work-up includes a full blood count with examination of the peripheral blood film, confirmation of the diagnosis by flow cytometry of peripheral blood, and molecular testing for TP53 aberrations (del(17p) by fluorescence in situ hybridisation and TP53 mutation by sequencing) together with immunoglobulin heavy-chain variable (IGHV) mutational status. These molecular results are decisive for treatment selection: TP53-aberrant disease responds poorly to fixed-duration venetoclax (Ven)-based regimens and favours continuous Bruton’s tyrosine kinase inhibitor (BTKi) therapy, whereas mutated IGHV predicts particularly durable remissions after fixed-duration Ven-obinutuzumab (Obi). Fitness assessment must therefore always be interpreted in parallel with molecular risk stratification rather than in isolation. In older adults, this baseline work-up should be accompanied by an ageing-focused evaluation – comorbidity burden, functional status, cognition, nutrition, polypharmacy, and social support – which forms the foundation for the structured, treatment-specific fitness assessment developed in the remainder of this review.

The contemporary first-line treatment landscape is defined by two principal paradigms: (1) continuous therapy with covalent BTKis – ibrutinib, acalabrutinib, or zanubrutinib – given until disease progression or intolerance; and (2) fixed-duration regimens combining the BCL-2 inhibitor Ven with a CD20 monoclonal antibody (Obi) or a BTKi for a defined period of 12–15 months [4, 5]. The landmark phase 3 CLL17 trial published online in the *New England Journal of Medicine* on December 6, 2025, definitively established the non-inferiority of both Ven-Obi and venetoclax-ibrutinib (Ven-Ibu) versus continuous ibrutinib in previously untreated CLL, with 3-year progression-free survival (PFS) rates of 81.1%, 79.4%, and 81.0%, respectively – while demonstrating substantially deeper remissions and lower discontinuation rates with the fixed-duration arms [6]. The evidence base for fixed-duration therapy also rests not on CLL17 alone but on the convergent findings of CLL14 (venetoclax-obinutuzumab [Ven-Obi] in unfit patients), GAIA-CLL13 (Ven combinations in fit patients), GLOW and CAPTIVATE (fixed-duration ibrutinib-venetoclax [I+V]), and AMPLIFY (fixed-duration acalabrutinib combinations), which together demonstrate the reproducibility of the fixed-duration benefit across fitness strata [3–5, 7–10]. Importantly, however, fixed-duration therapy is not universally preferred. Continuous BTKi therapy remains the optimal choice in defined settings – most notably TP53-aberrant disease (del(17p) and/or TP53 mutation), where time-limited Ven-based regimens yield inferior PFS, and in patients unable to undertake the intensive ramp-up monitoring that Ven requires.

This paradigm shift creates a critical new challenge: traditional fitness tools – the Cumulative Illness Rating Scale (CIRS), Eastern Cooperative Oncology Group Performance Status (ECOG-PS), and generic geriatric assessments – were used in the era of CIT and provide no guidance on the different safety and logistical demands of continuous BTKi versus fixed-duration Ven-based regimens [11, 12]. A patient who is an excellent candidate for continuous acalabrutinib may not be an appropriate candidate for Ven-Obi if they have severe renal impairment or high tumour burden and live far from a hospital capable of managing tumour lysis syndrome (TLS). Conversely, a patient with significant cardiac comorbidity and atrial fibrillation (AF) who would struggle on continuous ibrutinib may be ideally suited to Ven-Obi.

This review provides a treatment-specific fitness assessment framework for CLL in the targeted therapy era, drawing on current evidence from clinical trials, observational studies, and international guidelines. We propose a structured approach to matching patient fitness profiles to optimal treatment strategies, supported by recommendations for geriatric, cardiovascular (CV), and renal assessment, and conclude with suggested additions and critical appraisal of the existing evidence.

## Methods

This is a narrative review of the literature conducted to synthesise current evidence on fitness assessment in CLL in the era of targeted therapies. Searches were performed in PubMed, Scopus, and Embase, covering articles published from January 2015 to March 2026. Search terms included “chronic lymphocytic leukaemia,” “CLL,” “BTK inhibitor,” “venetoclax,” “fitness,” “frailty,” “geriatric assessment,” “cardiovascular risk,” “renal impairment,” “fixed-duration therapy,” and relevant combinations. Priority was given to phase 3 randomised controlled trials, prospective cohort studies, systematic reviews and meta-analyses, and current international guidelines from European Society for Medical Oncology (ESMO), American Society of Clinical Oncology (ASCO), and European Society of Cardiology (ESC). Case reports and small single-centre studies were excluded unless they specifically addressed an evidence gap relevant to the review. All selected references were reviewed by the authors for methodological quality and clinical relevance.

## The Two Treatment Paradigms and Their Distinct Fitness Demands

### Continuous BTKi Therapy

Continuous BTKi therapy involves daily oral administration until disease progression or intolerance. The principal fitness concerns for this strategy are CV comorbidity and adherence over an indefinite duration.

CV adverse events are a recognised class effect of all BTKis, though with differing frequencies: ibrutinib is associated with AF in up to 38% of patients, hypertension in approximately 25%, and rare but serious ventricular arrhythmias; acalabrutinib and zanubrutinib have substantially lower AF rates (4–9%) but comparable rates of hypertension [13, 14]. In the CLL17 trial, early discontinuation due to adverse events occurred in 33.2% of patients receiving continuous ibrutinib – the highest rate of the three arms – with CV events and infections as the primary drivers [6]. This underscores the importance of careful patient selection for continuous BTKi therapy.

From a logistical fitness perspective, continuous BTKi therapy requires only daily oral dosing, without the mandatory laboratory monitoring and hospital visits associated with Ven ramp-up. This makes it an appropriate choice for patients who live far from haematology centres, who have limited social support, or who have cognitive impairment affecting ability to manage complex monitoring schedules.

Drug-drug interactions are a critical fitness consideration for BTKis. Ibrutinib and acalabrutinib are predominantly CYP3A4 substrates; co-administration of strong CYP3A4 inhibitors (e.g., azole antifungals, clarithromycin) or inducers (e.g., rifampicin, phenytoin) may result in clinically significant alterations in drug exposure. Ibrutinib additionally interacts with warfarin and antiplatelet agents, complicating anticoagulation management in patients with AF or other thromboembolic indications [15].

### Fixed-Duration Ven-Based Regimens

Fixed-duration regimens combine Ven – a BCL-2 inhibitor – with a CD20 monoclonal antibody (Obi: Ven-Obi) and/or a BTKi (Ven-Ibu, Ven-acalabrutinib±Obi [AMPLIFY]) [5, 7, 9]. Treatment duration is typically 12 cycles (∼12–15 months), after which patients enter a treatment-free interval. The CLL17 trial demonstrated that this treatment-free interval is of substantial practical value: discontinuation due to adverse events or intercurrent illness occurred in only 14.6% of Ven-Obi patients versus 33.2% of continuous ibrutinib patients [6].

The principal fitness determinants for Ven-based regimens are renal function and tumour burden (which determine TLS risk), social support and geographic proximity to hospital (which determine feasibility of the intensive monitoring required during the ramp-up phase), and – for Ven-Obi – tolerance of intravenous infusions and Obi-associated infusion reactions. Renal impairment (creatinine clearance [CrCl]<80 mL/min) substantially increases TLS risk and requires dose modification protocols and enhanced monitoring [5, 16].

Patients receiving Obi undergo deep B-cell depletion, resulting in a higher short-term infection risk compared to BTKi monotherapy. In the CLL17 trial, serious adverse events were most frequent in the Ven-Obi arm (64.1% vs. 46.9% for Ven-Ibu and 47.7% for continuous ibrutinib), largely driven by infections, including grade ≥3 COVID-19 and other severe infections [6]. IgG levels should be checked at baseline in patients being considered for Ven-based regimens, and prophylactic antimicrobial and antiviral strategies discussed.

The biological rationale for fixed-duration therapy is the achievement of deep remissions, including undetectable minimal residual disease (uMRD), which allows sustained disease control in the absence of therapy. In CLL17, uMRD in peripheral blood was achieved in 73.3% of Ven-Obi and 47.2% of Ven-Ibu patients, versus 0% of continuous ibrutinib patients [6]. This depth of response, rather than continuous drug exposure, is the mechanism of durable disease control in the fixed-duration paradigm.

### The Role of Molecular Disease Features in Treatment-Specific Fitness Assessment

Fitness assessment cannot be considered in isolation from molecular disease characteristics. *TP53* mutation and/or del(17p) are particularly relevant: ESMO guidelines recommend BTKi-based therapy as the preferred first-line approach for patients with *TP53*-aberrant CLL, given the inferior outcomes observed with fixed-duration Ven-based regimens in this subgroup [3]. *IGHV* mutational status also informs treatment selection, with mutated *IGHV* patients achieving particularly durable remissions with fixed-duration Ven-Obi [3]. Fitness assessment should therefore always occur in parallel with, not in isolation from, molecular risk stratification. Table 1 sets out the principal molecular disease features, their prognostic and predictive implications, and the corresponding treatment considerations, so that disease biology and treatment selection can be read side by side.

**Table 1.** Molecular disease features and their treatment implications in CLL

Molecular feature	Prognostic/predictive implication	Preferred treatment consideration	Fitness interpretation
TP53 aberration (del(17p) and/or TP53 mutation)	Highest-risk marker; short remissions and inferior PFS with fixed-duration Ven-based regimens	Continuous BTKi preferred (ESMO); fixed-duration Ven generally avoided as sole strategy	Even a fully fit patient with TP53 aberration is steered towards continuous BTKi; disease biology overrides a favourable fitness profile
Unmutated IGHV	Historically adverse with CIT; largely overcome by targeted agents	Both paradigms effective; fixed-duration Ven and continuous BTKi both appropriate	Fitness domains (cardiac vs. renal/TLS/logistics) become the main discriminators between the two paradigms
Mutated IGHV	Favourable; particularly durable remissions with fixed-duration Ven-Obi	Fixed-duration Ven-Obi especially attractive where feasible	A fit patient with mutated IGHV is an ideal candidate for a time-limited regimen with a treatment-free interval
Complex karyotype/other high-risk cytogenetics	Associated with shorter PFS across regimens; refines risk beyond TP53	Individualised; continuous BTKi often favoured in the highest-risk profiles	Supports closer monitoring and may lower the threshold for continuous therapy irrespective of fitness

BTKi, Bruton’s tyrosine kinase inhibitor; FISH, fluorescence in situ hybridisation; IGHV, immunoglobulin heavy-chain variable region; PFS, progression-free survival; TLS, tumour lysis syndrome.

## A Treatment-Specific Fitness Assessment Framework

Tables 2 and 3 provide a systematic summary of trial-level evidence supporting this treatment-specific fitness assessment framework. Table 2 summarises the impact of fitness domains on BTKi treatment outcomes across eight key studies, demonstrating that ECOG-PS ≥2 is the most consistent marker of poorer outcomes, while CIRS >6 is more variably associated with tolerability and discontinuation. Table 3 presents corresponding evidence for Ven-based regimens, where CIRS-defined fit/unfit status appears less predictive of efficacy and geriatric impairment can improve with treatment. This framework should guide individualised treatment selection after initial haematologist assessment. The recommendations of the major international societies (ESMO, ASCO, ESC, and British Society for Haematology [BSH]) relevant to fitness assessment and treatment selection are summarised in Table 4. Because the two treatment paradigms differ most consequentially in their toxicity profiles, a direct comparison of the principal toxicities of continuous BTKi therapy versus fixed-duration Ven-based regimens, and their implications for fitness, is presented in Table 5.

**Table 2.** Impact of fitness on treatment outcomes of BTKis

Trial [ref]	*N*	Regimen	Population	Key outcome	Fitness impact
Swedish RWE [17]	95	Ibrutinib	Real-world unfit; median CIRS 6; 45% CIRS ≥6	CIRS ≥6: significantly shorter PFS and OS vs. CIRS <6	CIRS ≥6 independently predicted PFS and OS on multivariate analysis
RESONATE-2 [18, 19]	269	Ibrutinib vs. Clb	Elderly (≥65 yrs); del(17p) excluded; median age 73	7-yr PFS 59% vs. 9%; 9-yr OS 68% (ibrutinib arm)	Age alone did not predict PFS; efficacy maintained in patients ≥75 yrs and across varying comorbidity levels
ECOG1912 [20]	529	Ibrutinib + rituximab vs. FCR	Fit: age ≤70, ECOG 0–2; median age 57	3-yr PFS 89.4% (IR) vs. 72.9% (FCR); OS HR 0.47 (*p* = 0.018) at 6 yrs	CIRS independently predicted ibrutinib discontinuation (HR 1.13/unit, *p* = 0.009); age was not an independent predictor
ELEVATE-TN [21]	535	Acalabrutinib±Obi vs. G-Clb	Unfit: age ≥65 or CIRS >6, CrCl <70; median age 70	5-yr PFS: acalabrutinib 62.5% vs. G-Clb 23.0% (*p* < 0.0001)	Fitness was not a differential predictor within acalabrutinib arms; AF 5–8% vs. 16% with ibrutinib
Italian EVIdeNCE RWE [22]	309	Ibrutinib	Unselected; median age 71; 60% aged ≥70; ECOG 0–1 in 90%	2-yr retention 70.2%; CIRS >6 associated with shorter PFS in elderly	CIRS >6 independently reduced treatment tolerance and PFS, with stronger effect in patients >70 yrs
Romanian RWE [23]	123	Ibrutinib (any line)	Unselected; median age 65	Median PFS 50 mo; ORR 86.2%	ECOG-PS ≥2 independently predicted shorter PFS; CIRS ≥6 predicted higher toxicity-related discontinuation
ALPINE [24]	652	Zanubrutinib vs. ibrutinib	Unselected; median age 67	PFS HR 0.65 (*p* = 0.002) favouring zanubrutinib; AF: 5.2% vs. 10.1% (*p* = 0.02)	Cardiac history predicted ibrutinib-specific AF; zanubrutinib benefit preserved across risk groups
US Community RWE [25]	2,815	Ibrutinib vs. acalabrutinib vs. zanubrutinib	ECOG 0–1 in 62–66%; median age 71–72	TTD: ibrutinib 13.7 mo, acalabrutinib 19.2 mo, zanubrutinib 19.3 mo	Second-generation BTKi advantage maintained across all fitness strata; cardiac comorbidities predicted ibrutinib-specific CV events

AF, atrial fibrillation; BTKi, Bruton’s tyrosine kinase inhibitor; CIRS, Cumulative Illness Rating Scale; Clb, chlorambucil; CrCl, creatinine clearance; CV, cardiovascular; ECOG-PS, Eastern Cooperative Oncology Group Performance Status; FCR, fludarabine-cyclophosphamide-rituximab; G-Clb, obinutuzumab-chlorambucil; HR, hazard ratio; IR, ibrutinib-rituximab; Obi, obinutuzumab; ORR, overall response rate; OS, overall survival; PFS, progression-free survival; RWE, real-world evidence; TTD, time to discontinuation.

**Table 3.** Impact of fitness domains on treatment outcomes of Ven-based regimens

Trial [ref]	*N*	Regimen	Population	Key outcome	Fitness impact
MURANO [26]	389	Ven-R vs. BR	Mixed; no formal CIRS stratification; median age 65; ECOG ≥2 excluded	5-yr PFS 53.3% (Ven-R) vs. 17.3% (BR); OS HR 0.40 (*p* < 0.001)	No formal fitness stratification; ECOG 0–1 required for enrolment, limiting conclusions in unfit populations
CLL14 [5, 27]	432	Ven-Obi vs. Clb-Obi	Unfit: CIRS >6 and/or CrCl <70; median age 72; median CIRS 8	6-yr median PFS 76.2 mo (Ven-Obi) vs. 36.4 mo (Clb-Obi), HR 0.40; 5-yr OS 81.9% vs. 77.0% (NS)	Enrolled exclusively unfit patients; high CIRS did not impair remission depth (uMRD 70%)
GLOW [8, 10]	211	I + V vs. G-Clb	Unfit: age ≥65 and/or CIRS >6 or CrCl <70; median age 71	66-mo PFS: I+V 51.7% vs. G-Clb 18.1%, HR 0.273 (*p* < 0.0001)	I+V superior in all pre-specified unfit subgroups; frailty (by CGA) improved in 53% on treatment
HOVON139/GiVe [28]	67	Ven-Obi (unfit, CGA-assessed)	FCR-ineligible; CGA-assessed; median age 71	uMRD comparable to CLL14; proportion with ≥2 geriatric impairments fell from 61% to 36%	First study to demonstrate treatment-induced frailty improvement with targeted therapy using CGA
CLL13/GAIA [7]	926	Ven-Obi±ibrutinib vs. CIT vs. Ven-R	Fit: CIRS ≤6, CrCl ≥70; median age 61	PFS HR 0.32 (Ven-Obi+Ibru vs. CIT, *p* < 0.000001); uMRD at 15 mo: triple 92.2%, doublet 86.5%, CIT 52%	Enrolled fit patients; fitness influenced tolerability within each arm rather than cross-arm efficacy
CLL13+CLL14 Pooled [29]	410	Ven-Obi (fit vs. unfit)	Fit: CIRS ≤6, CrCl ≥70; unfit: CIRS >6 and/or CrCl ≤70	3-yr PFS: unfit 86.4% vs. fit 87.5% (NS); uMRD: unfit 80.3% vs. fit 85.1% (NS)	CIRS-based fitness did not predict PFS or OS; fitness-based stratification may no longer apply to Ven-Obi
CLL-Frail [30]	43	Acalabrutinib (frail, age ≥80)	Frail: age ≥80 and/or CGA-confirmed frailty; median age 81	ORR 79.1%; PFS and OS comparable between frail and non-frail	First prospective trial in frail CLL; BTKi safe in frail patients; frailty improved in 53% during treatment

BR, bendamustine-rituximab; CGA, comprehensive geriatric assessment; CIRS, Cumulative Illness Rating Scale; CIT, chemoimmunotherapy; Clb, chlorambucil; CrCl, creatinine clearance; G-Clb, obinutuzumab-chlorambucil; HR, hazard ratio; I+V, ibrutinib-venetoclax; NS, not statistically significant; Obi, obinutuzumab; ORR, overall response rate; OS, overall survival; PFS, progression-free survival; *R*, rituximab; uMRD, undetectable minimal residual disease; Ven, venetoclax; FCR, fludarabine-cyclophosphamide-rituximab.

**Table 4.** Summary of major society recommendations relevant to fitness assessment and treatment selection in CLL

Society/guideline	Key recommendations relevant to fitness and treatment selection
ESMO (2024 interim update)	Targeted agents preferred over CIT for all fitness groups; continuous BTKi preferred for TP53-aberrant disease; fixed-duration Ven-Obi endorsed including in unfit patients
ASCO (geriatric oncology guideline)	Recommends structured geriatric assessment for older adults receiving systemic therapy; identifies vulnerabilities in function, comorbidity, cognition, and nutrition
ESC (2022 cardio-oncology guideline)	Baseline CV risk stratification before BTKi; blood pressure control and rhythm surveillance during therapy; cardio-oncology referral for high-risk patients
BSH (good-practice guidance)	Emphasises individualised, comorbidity-informed treatment selection, and supportive care, including infection prophylaxis and immunoglobulin monitoring

ASCO, American Society of Clinical Oncology; BSH, British Society for Haematology; BTKi, Bruton’s tyrosine kinase inhibitor; ESC, European Society of Cardiology; ESMO, European Society for Medical Oncology.

**Table 5.** Comparative toxicity profile of continuous BTKi vs. fixed-duration Ven-based regimens and implications for fitness

Toxicity domain	Continuous BTKi therapy	Fixed-duration Ven-based therapy
CV	Class-effect AF (up to 38% ibrutinib; 4–9% 2nd-generation) and hypertension (∼20–25%); rare ventricular arrhythmia	Low intrinsic cardiotoxicity; Obi infusion reactions may stress patients with cardiac disease; residual BTKi risk in Ven-Ibu
TLS	Not applicable; no ramp-up required	Principal early hazard; risk rises with tumour burden and renal impairment, requiring staged ramp-up and monitoring
Infection	Chronic humoral suppression; cumulative risk over indefinite exposure	Higher short-term risk with Obi-driven B-cell depletion; time-limited exposure thereafter
Haematological	Neutropenia, bleeding tendency (platelet dysfunction)	Neutropenia (notably with Obi); manageable with growth factors and dose modification
Treatment burden/discontinuation	Indefinite daily dosing; higher long-term discontinuation (33.2% in CLL17); adherence-dependent	Defined 12- to 15-month course; lower discontinuation (14.6% Ven-Obi in CLL17); intensive early monitoring and then treatment-free interval
Dominant fitness implication	CV comorbidity and long-term adherence capacity	Renal function, tumour burden, and social/logistical support for ramp-up

BTKi, Bruton’s tyrosine kinase inhibitor; CLL17, phase 3 trial of fixed-duration vs. continuous therapy; Ven-Ibu, venetoclax-ibrutinib; Ven-Obi, venetoclax-obinutuzumab.

### Core Assessment Approach

Irrespective of the intended treatment, the initial assessment should be performed by the treating haematologist and include full medication review with particular attention to CYP3A4 modulators and anticoagulants; screening for CV comorbidity and risk factors; baseline renal function (eGFR); serum IgG level; and frailty/resilience screening by using screening tools like Geriatric 8 (G8) or Canadian Study of Health and Aging Clinical Frailty Scale (CSHA-CFS). Following this initial evaluation, treatment-specific further assessment should be done (as outlined in Tables 2, 3 and the proposed clinical pathway).

A key principle is that the inability to access one or more recommended assessments should not prevent treatment. The framework is designed for best practice; where specialist referral is not available in a timely manner and the patient requires treatment, the haematologist should proceed with the best available information and document the clinical rationale.

## Geriatric Assessment and Frailty (Lower Resilience)

### Frailty in CLL: Prevalence and Definition

Frailty – or lower resilience – is defined as a clinical syndrome of increased susceptibility to stressors associated with reduced physiological reserve [31]. It is present in a substantial proportion of older adults with CLL, with the majority demonstrating at least one geriatric impairment on structured assessment even when ECOG-PS is 0 or 1 [28, 31]. The Fried phenotypic model defines frailty as the presence of three or more of unintentional weight loss, exhaustion, weakness, slow gait, and decreased physical activity [32]. The Rockwood deficit accumulation model quantifies frailty as a continuous variable [33].

### Frailty, Targeted Therapy, and the Dynamic Nature of Resilience

A paradigm-shifting observation from recent data is that frailty is not static and may be partially disease-mediated in CLL – and therefore potentially reversible with effective therapy. In the HOVON139/GiVe trial, geriatric burden decreased significantly during fixed-duration Ven-Obi treatment in older, fludarabine-cyclophosphamide-rituximab-unfit patients, with more than half of those with an initially high geriatric burden converting to a low burden [28]. Similarly, the 3-month ibrutinib lead-in used in the Ven-Ibu (GLOW, CAPTIVATE) regimens typically improves patient resilience prior to Ven addition [10].

A prospective cohort study by Martino et al. [34] involving 82 older adults (median age 75 years) receiving BTKi or BCL-2 inhibitor therapy demonstrated that the CSHA-CFS score above 3 independently predicted treatment-related toxicity and discontinuation, and was associated with shorter overall survival (OS). Importantly, this study used a simple, rapid frailty tool – the CFS – which is more practical in busy haematology settings than comprehensive geriatric assessment (CGA). The clinical implication is twofold: firstly, frailty alone should not preclude patients from receiving targeted therapy, including fixed-duration combination regimens; secondly, re-assessment of frailty during treatment is essential, as improvement in resilience may allow escalation or modification of therapy.

### Recommended Geriatric Screening Tools

Table 6 summarises the recommended geriatric screening tools and their characteristics. The G8 tool is the recommended first-line screening instrument; a score of ≤14 indicates high risk of lower resilience and warrants further evaluation [35, 36]. The CSHA-CFS is particularly valuable in high-volume haematology settings as it can be administered in under 2 min at the bedside [34]. Where a specialist geriatrician is available, patients scoring ≤14 on G8 should be referred for CGA before a final treatment decision is made [36].

**Table 6.** Geriatric screening tools for use in patients with CLL

Tool	Domains	Time (min)	Recommendation
G8	Age, weight loss, BMI, motor, psychological, medications, self-rated health	∼5	Preferred first-line screening tool
VES-13	Age, self-rated health, functional limitations and disabilities	∼5 (self)	Alternative to G8
GAH Scale	Medications, gait, mood, ADL, nutrition, comorbidities	10–12	Useful where geriatrician unavailable
CSHA-CFS	Clinical frailty 1–9 scale	<2	Rapid bedside tool; CFS >3 predicts BTKi toxicity

ADL, activities of daily living; CFS, Clinical Frailty Scale; CLL, chronic lymphocytic leukaemia; CSHA, Canadian Study of Health and Aging; G8, Geriatric 8; GAH, Geriatric Assessment in Haematology; VES-13, Vulnerable Elders Survey-13.

## CV Assessment

### BTKi Class-Effect Cardiotoxicity

CV adverse events are a recognised class effect of BTKi therapies, with the highest rates observed with ibrutinib. A state-of-the-art review documented ibrutinib-associated AF in up to 38% of patients and a more than 4-fold increase in cardiac events versus untreated CLL patients [13]. Hypertension occurs in approximately 20–25% of patients receiving any BTKi, and ventricular arrhythmias, though rare, carry a risk of sudden cardiac death estimated at approximately 1 per 1,000 person-years [13].

Second-generation BTKis demonstrate improved kinase selectivity and meaningfully lower rates of AF: 4–9% for acalabrutinib and zanubrutinib versus up to 38% for ibrutinib in head-to-head comparisons [13, 14]. However, CV toxicity is not eliminated with second-generation agents, and hypertension rates are comparable across the class [13]. A meta-analysis of 45 published studies found an overall AF incidence of 5% across all BTKis, with substantially lower rates for second-generation drugs [37].

The mechanism is multifactorial, involving off-target inhibition of TEC and PI3K-delta kinases, disruption of cardiac ion channels including PI3K-dependent regulation, and activation of profibrotic signalling pathways promoting AF. Emerging evidence suggests that genetic variants in KCNQ1 and GATA4 may predict individual susceptibility to BTKi cardiotoxicity, though clinical implementation awaits validation in larger prospective cohorts [13].

### Risk Stratification and Monitoring

All patients considered for BTKi therapy should undergo structured CV risk stratification prior to treatment. High-risk conditions include uncontrolled hypertension, active or prior arrhythmias (including AF), heart failure, cardiomyopathy, severe valvular heart disease, and coronary artery disease [15, 38]. For patients at low CV risk, haematologist-led follow-up with structured home blood pressure monitoring (weekly in the first month, then every 2–3 weeks) is appropriate. Patients at high risk should be referred to a cardiologist or cardio-oncology service before treatment initiation [15].

For Ven-based regimens, CV assessment is directed primarily at tolerance of Obi infusion-related reactions (which can cause hypotension, tachycardia, and dyspnoea – particularly problematic in patients with pre-existing cardiac disease) and, for Ven-Ibu combinations, at the cumulative CV effect of the BTKi component [15]. The ESMO guideline notes that I+V should be considered carefully in older patients with cardiac comorbidities, suggesting preference for acalabrutinib or Ven-Obi in this group [3].

Blood pressure should be controlled to below 160/100 mmHg before initiating any BTKi, with a treatment target below 130/80 mmHg. Opportunistic AF screening by ECG rhythm strip or pulse palpation at each clinic visit during BTKi therapy is recommended, with any suspected AF confirmed by 12-lead ECG or Holter monitoring [15].

## Renal Assessment

Renal impairment is present in approximately 24% of newly diagnosed CLL patients and is an independent adverse prognostic factor associated with significantly shorter OS [39]. Mechanisms include age-related nephron loss, direct CLL infiltration, glomerular diseases, and obstructive uropathy [40].

The clinical relevance of renal assessment differs markedly between the two therapeutic paradigms. For continuous BTKi therapy, renal impairment generally does not preclude treatment, though dose adjustments may be required for some agents; the main concern is polypharmacy-related nephrotoxicity [15]. For Ven-based regimens, renal function is a critical determinant of TLS risk: CrCl below 80 mL/min substantially increases TLS risk during the mandatory ramp-up phase, requiring more intensive monitoring, enhanced hydration protocols, and in some cases, modified ramp-up schedules [5, 16]. Nephrology referral is generally recommended for patients with eGFR below 30 mL/min/1.73 m^2^ per KDIGO guidelines, but should be considered before Ven initiation in those with CrCl below 80 mL/min [16, 41].

In the CLL14 and GLOW trials, Ven-based regimens were successfully administered in patients with CrCl as low as 30 mL/min with enhanced monitoring protocols, demonstrating that renal impairment is a factor requiring careful management rather than an absolute contraindication [5, 10]. Longitudinal renal monitoring during treatment is recommended, as renal function may change dynamically during therapy.

## Infection Risk Assessment and Immunological Support

Patients with CLL are inherently immunosuppressed as a consequence of the disease – with progressive hypogammaglobulinaemia affecting up to 85% during the disease course – and its treatments [42]. Infections account for 30–60% of CLL-related mortality, and this risk is amplified during active therapy, particularly with Ven-based regimens involving anti-CD20 antibodies [42, 43].

### Immunoglobulin Monitoring and Replacement Therapy

Routine IgG testing is recommended in all patients with CLL [42]. Immunoglobulin replacement therapy is indicated for patients with recurrent or severe infections and IgG levels below 500 mg/dL, though international guidelines vary in their specific thresholds [42, 44]. A real-world study by Soumerai et al. [44] demonstrated that immunoglobulin replacement therapy was associated with significant reductions in infections, severe infections, and antimicrobial use in CLL patients with hypogammaglobulinaemia. Baseline IgG is particularly important before initiating Ven-Obi, given the depth of B-cell depletion and associated infection risk demonstrated in CLL17 [6].

### Vaccination and Prophylaxis

All patients with CLL should be encouraged to receive recommended vaccinations – including influenza, COVID-19, pneumococcal disease, herpes zoster, RSV, tetanus, and hepatitis B – prior to initiating CLL-directed therapy where possible, as vaccination responsiveness is reduced during treatment [45]. Live vaccines are contraindicated during active therapy. Prophylactic antimicrobial and antiviral strategies (e.g., antiviral prophylaxis for herpes zoster with Ven, PJP prophylaxis per institutional protocols) should be discussed with patients beginning fixed-duration regimens.

## Proposed Clinical Pathway for Treatment-Specific Fitness Assessment

Figure 1 presents a proposed fitness-guided clinical algorithm for first-line treatment selection in CLL. The pathway is designed to be used sequentially at the point of treatment initiation and integrates the fitness domains discussed throughout this review into a practical decisional tool. Patients in whom all four questions are negative represent a group in whom both continuous BTKi and fixed-duration Ven-based therapy are clinically appropriate; in this group, *IGHV* mutational status and patient preference become the primary determinants of the final decision. The algorithm concludes with treatment-specific pre-treatment checks and a continuous monitoring framework applicable throughout the course of therapy. It should be emphasised that this pathway represents best-practice guidance rather than a rigid protocol; lack of access to any individual assessment should not delay or prevent treatment. We stress that this algorithm is a conceptual tool derived from expert synthesis of the current evidence and is not itself prospectively validated; it is intended to structure clinical reasoning and to serve as a hypothesis for future validation studies, and should not be interpreted as a validated decision instrument.

**Fig. 1.**
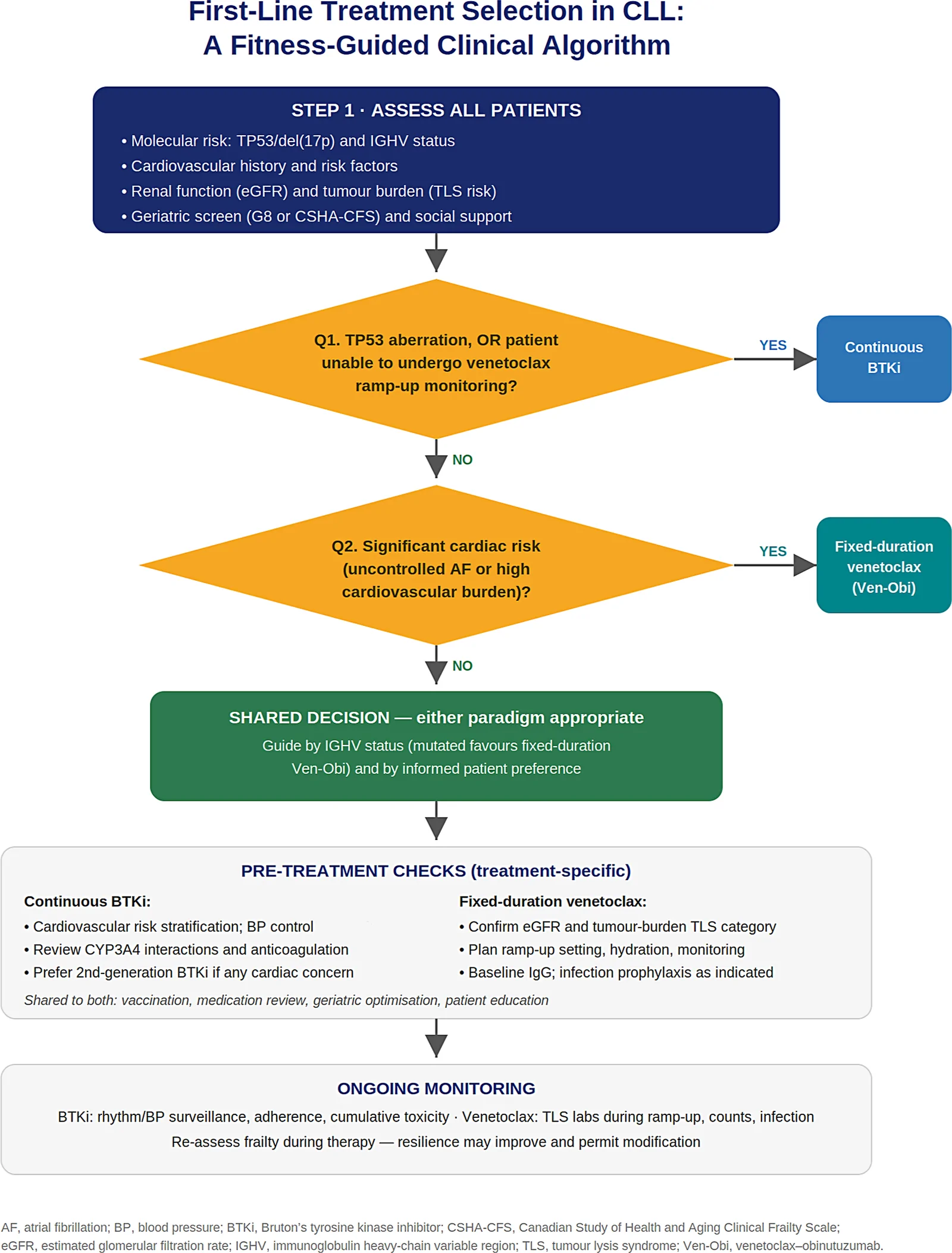
Treatment selection in CLL: a fitness-guided clinical algorithm. Following universal baseline assessment (step 1: molecular risk, CV, renal, and geriatric screening), two sequential fitness-directed questions guide allocation. Q1 identifies patients for whom continuous BTKi is preferred (TP53 aberration or inability to undergo Ven ramp-up monitoring); Q2 identifies patients for whom fixed-duration Ven-Obi is preferred (significant cardiac risk). Patients with no dominant fitness concern proceed to a shared decision guided by IGHV status and patient preference. Treatment-specific pre-treatment checks and ongoing monitoring apply thereafter. This is a conceptual, expert-derived tool that has not been prospectively validated.

## Patient Perspective and Shared Decision-Making

Patient-centred care is integral to fitness assessment and treatment selection. Survey data from the 2023 Global Leukaemia Experience Survey demonstrated that only 43% of patients reported receiving clear information about treatment side effects, and fewer than half felt sufficiently involved in initial treatment decisions [46]. This represents a critical and modifiable gap.

Patient preferences for treatment modality are meaningful and should actively inform clinical decisions. Approximately 70% of patients prefer oral therapies, and most express a preference for fixed-duration treatment when it is explained clearly – particularly the prospect of a treatment-free interval [46, 47]. However, the logistical complexity of Ven ramp-up, including mandatory laboratory monitoring and hospital visits, must be communicated clearly, as these requirements may be prohibitive for some patients.

The concept of “fitness” should be communicated to patients as “suitability for treatment” or “medical fitness,” as the conventional frailty terminology may be experienced as stigmatising, particularly given that many patients feel unwell prior to diagnosis and may not consider themselves fit in any sense [11]. Age should not be presented as a barrier to treatment; patients should understand that the goal of fitness assessment is to find the safest, most effective treatment for them as an individual.

## “Health System Fitness”: Access, Affordability, and Infrastructure

The framework proposed here assumes ready access to both BTKis and Ven, but this assumption does not hold uniformly. Across parts of Asia, Africa, and Latin America, availability and affordability remain uneven: not all BTKis or Ven are approved in every country, and even where they are approved, reimbursement pathways may be slow and out-of-pocket costs prohibitive in health systems without universal coverage. Ven ramp-up further requires reliable laboratory monitoring and hospital capacity to manage TLS, infrastructure that is not uniformly available outside major urban centres. As a result, although international guidelines recommend targeted therapies as standard, CIT (e.g., bendamustine-rituximab) remains widely used in resource-limited settings on grounds of cost and access. A comprehensive assessment of suitability must therefore incorporate not only biological and clinical fitness but also “health system fitness” – drug access, reimbursement, geographic proximity to appropriately resourced centres, and the infrastructure needed for safe Ven ramp-up. Recent regional standardisation efforts, such as the Delphi consensus process reported by Martino et al. [48], illustrate how expert frameworks can be adapted to local resource realities, and future iterations of fitness assessment should be explicitly contextualised to the health system in which they are applied [48, 49].

## Research Gaps and Future Directions

Several important evidence gaps remain in the field of fitness assessment for targeted therapy in CLL: firstly, no prospective randomised study has evaluated whether fitness-directed treatment selection – specifically, using pre-defined fitness criteria to allocate patients to continuous BTKi versus fixed-duration Ven-based therapy – improves outcomes. Such trials are urgently needed. Concretely, we propose a biomarker-stratified randomised design in which treatment-naïve patients are first classified by a pre-specified, multidomain fitness algorithm (integrating CV risk, renal function, tumour burden, and a validated frailty screen such as the G8 or CSHA-CFS) and then randomised, within each fitness stratum, either to fitness-directed allocation or to physician’s choice. Co-primary endpoints would be PFS and a composite tolerability endpoint (grade ≥3 toxicity-free survival and treatment discontinuation rate), with pre-planned subgroup analyses by TP53/IGHV status, patient-reported outcomes, and health economic measures. A pragmatic, registry-embedded design would improve generalisability and feasibility, and international collaboration would be required to reach adequate power within each stratum.

Secondly, the geriatric assessment tools currently recommended for CLL (G8, VES-13, GAH) have not been prospectively validated for prediction of adverse outcomes specifically with BTKis or BCL-2 inhibitors. Validation studies in contemporary trial populations are required [11].

Thirdly, long-term sequencing data after fixed-duration therapy are still maturing. Optimal strategies for managing CLL progression after a fixed-duration regimen – including feasibility and depth of re-treatment – will influence when fixed-duration therapy is most appropriate [4].

Fourthly, MRD-guided treatment duration – in which therapy is discontinued upon achieving uMRD rather than after a fixed number of cycles – is an area of active investigation that may further personalise the fitness demands of therapy [3]. The UK phase 3 FLAIR trial has already demonstrated that MRD-guided I+V, in which treatment duration is set at twice the time taken to reach uMRD, improves PFS and OS relative to fludarabine-cyclophosphamide-rituximab, establishing proof of principle for response-adapted duration [50]. Ongoing MRD-guided and MRD endpoint studies (including the Ven-based arms of GAIA-CLL13 and successor trials) are expected to refine how depth of response can be used to individualise both the intensity and the duration of therapy, with direct implications for the monitoring burden that a given patient must be fit to sustain.

## Conclusion

The era of targeted therapies in CLL demands a fundamental reconceptualisation of patient fitness assessment – one that is treatment-specific, multidimensional, dynamic, and patient-centred. The binary “fit/unfit” classification of the CIT era is no longer adequate. Modern fitness assessment must distinguish the fitness demands of continuous BTKi therapy (dominated by CV risk and long-term adherence capacity) from those of fixed-duration Ven-based regimens (dominated by renal function, tumour burden, TLS risk, and social support for intensive monitoring).

The landmark CLL17 trial has established that fixed-duration Ven-based therapy is non-inferior to continuous ibrutinib in terms of PFS, with superior MRD clearance and lower treatment discontinuation – particularly in older patients. These findings position fixed-duration therapy as the preferred option for most patients in whom it is logistically and clinically feasible, provided their fitness profile is compatible with the specific demands of the ramp-up phase. Continuous BTKi therapy nonetheless remains preferable in TP53-aberrant disease and where Ven monitoring is not feasible. Critically, the screening tools that underpin this framework – including the G8 and CSHA-CFS – remain unvalidated for outcome prediction in the BTKi and BCL-2 inhibitor era; their prospective validation is the single most important step required before treatment-specific fitness assessment can be regarded as evidence-based rather than expert-derived.

Most patients with CLL – including those with lower resilience – are candidates for targeted therapy. The clinical imperative is not to determine whether a patient “qualifies” for treatment, but to identify which treatment best matches their individual fitness profile. Structured, treatment-specific fitness assessment, embedded within a shared decision-making framework centred on the patient, is the standard of care for CLL in 2026.

## Acknowledgments

This manuscript is original work and has not been submitted or under consideration for publication elsewhere. All authors have reviewed and approved the manuscript.

## Conflict of Interest Statement

The authors have no known competing interests or conflict of interest to declare.

## Funding Sources

Open-access publication of this article was funded by Qatar National Library (QNL), Doha, Qatar. QNL had no role in the conception, design, or planning of this review; in the identification, selection, analysis, or interpretation of the literature; in the writing of the manuscript; or in the decision to submit it for publication. Its support was limited to covering article processing charges. No other funding, grant, or sponsorship was received for this work.

## Author Contributions

All authors made a significant contribution to this work. Dr. Yousef Al-Asa’d: writing – original draft. Dr. Salem H. Alshemmari and Dr. Afaf Al Battah: supervision. Dr. Nabeel Qasem: writing – review and editing. Dr. Mohamed A. Yassin: conceptualisation and supervision.
